# Dietary Challenges in Children with Gluten-Related Disorders: A Study on Food Neophobia

**DOI:** 10.3390/nu16223924

**Published:** 2024-11-17

**Authors:** Julyana Nogueira Firme, Emanuele Batistela dos Santos, Renata Puppin Zandonadi, Eduardo Yoshio Nakano, Raquel Braz Assunção Botelho

**Affiliations:** 1 Human Nutrition Graduate Program, University of Brasília, Brasília 70910-900, Brazil; julyanafirme@gmail.com; 2 Department of Food and Nutrition, Federal University of Mato Grosso, Cuiabá 78060-900, Brazil; emanuelebatistela.ufmt@gmail.com; 3 Nutrition Department, University of Brasília, Brasília 70910-900, Brazil; raquelbotelho@unb.br; 4 Department of Statistics, University of Brasília, Brasília 70910-900, Brazil; nakano@unb.br

**Keywords:** food neophobia, gluten-related disorder, eating behavior, celiac disease, child

## Abstract

Background/Objectives: Food neophobia (FN) in childhood is characterized by resistance to new foods, potentially impairing health and diet quality and impacting physical and mental development. Adequate nutrition in early childhood is crucial to preventing future problems. FN demands special attention, especially in cases of gluten-related disorders (GRD), due to the risks associated with restrictive diets and food allergies. The objective of this study was to investigate and classify the prevalence of FN among Brazilian children aged 4 to 11 years with GRD. Methods: For this cross-sectional study, a questionnaire validated in Portuguese, with 25 items, assessed FN in three domains: general FN, FN of fruits, and FN of vegetables. Data were collected via Google Forms. Results: Of 209 children with GRD, the majority were female, 83.7% were diagnosed with celiac conditions, 81.3% followed a diet, and 43.1% had moderate total food neophobia. Brazilian children with GRD have a moderate prevalence of food neophobia. General and vegetable neophobia predominate, while fruit FN is lower. Girls exhibit greater neophobia towards vegetables. Conclusions: Neophobia does not vary with age, suggesting the persistence of the behavior and potential nutritional challenges in adult life. Differentiated attention to this population is essential to minimize long-term impacts.

## 1. Introduction

Food neophobia (FN) is a disorder that frequently occurs in childhood and is defined as a behavior related to the reluctance to eat new foods and accept previously introduced flavors or flavors that have a different consistency [1]. FN plays a significant role in determining food preferences and choices, significantly affecting the diet’s quality [2]. Adverse effects of FN on children’s daily food intake include an increase in high-calorie and low-nutrient foods [3,4]. By consuming a smaller amount and variety of fruits and vegetables, children with neophobic behavior are more likely to be overweight [3,4]. The lack of diversity in the diet caused by FN limits the amount of nutrients ingested to keep the body healthy [5]. In addition, a child’s cognitive and physical capabilities can be impaired when there is a severe and/or persistent imbalance, as it can impact multiple body systems, including the neurological system [6].

In this sense, FN is a behavior that requires special care from parents, caregivers, family members, educators, and health professionals, as this behavior can cause eating disorders throughout life, in addition to harming the child’s biopsychosocial development [7]. Individuals with a high FN can become a specific group of consumers, given the sensory aversions and fear when consuming unknown and/or considered non-secure foods [8]. FN is common among children aged 2 to 10 years. However, it can be accentuated in children with very restrictive diets, such as food allergies and intolerances [9].

Among the health conditions that require restrictive diets as treatment, gluten-related disorders (GRD) stand out. GRD are chronic conditions, with or without autoimmune origin, caused by constant gluten exposure in cereals such as rye, barley, and wheat [10]. GRD include celiac disease (CD), non-celiac gluten sensitivity, gluten ataxia, dermatitis herpetiformis, and wheat allergy [10]. Based on global prevalence, the National Federation of Celiac Associations in Brazil (Fenacelbra) highlights that the celiac condition affects approximately two million Brazilians, with 80% not having a known diagnosis and 70% only becoming aware of it after the age of 20 [11].

The FN in GRD individuals is little studied, but it is assumed to be higher than in the general population as a defensive way to protect the body from unsafe or dangerous foods [8]. Therefore, we hypothesize that the prevalence of FN in Brazilian GRD children is significantly higher compared to the general Brazilian pediatric population because the dietary restrictions imposed by CD may contribute to the development or worsening of neophobic behavior about food, with possible implications for the diet’s quality, nutritional health, and biopsychosocial development of these children.

In a gluten-free diet (GFD), besides ensuring gluten restriction, one of the focal points of GRD treatment is maintaining nutritional balance to meet energy and nutritional needs, allowing children to grow and develop. Therefore, expanding knowledge about FN in GRD patients is necessary to provide better health support and nutritional guidance for this group [8], especially in children, as children with diets that require food restrictions demonstrate eating difficulties, with negative consequences on the dynamics of meals, growth, and development [4]. Therefore, the study aimed to investigate and classify the prevalence of FN among Brazilian children aged 4 to 11 years with GRD.

## 2. Materials and Methods

A cross-sectional exploratory study was performed in two stages: (i) application of the questionnaire to identify FN in GRD Brazilian children aged 4 to 11 years with a previously validated questionnaire [3]; and (ii) data analysis to characterize the national prevalence of FN in children with GRD compared with children without GRD, described in a previous study [12]. The study was approved by the Health Sciences Ethics Committee of the University of Brasília, n° 4.407.816, and followed the guidelines established by the Declaration of Helsinki.

### 2.1. Sample

The sample comprises caregivers of GRD children who agreed to participate in the study and knew the child’s eating habits. A caregiver was considered the adult responsible for looking after the child, such as a mother, father, grandmother, brother/sister, or uncle. According to previous instructions, only one of the caregivers could participate in the research by answering the questionnaire. The study included only the participants who answered that a physician diagnosed the child with GRD. Also, the caregiver had to answer what type of GRD the child presents.

From May 2021 to May 2023, with convenience and non-probabilistic sampling, the snowball sampling technique was used to recruit respondents through social media, such as Instagram^®^, Facebook^®^ Groups, quick messaging applications, and email.

In addition, entities focused on GRD patients helped to spread the search, such as the National Federation of Celiac Associations in Brazil (FENACELBRA); Brazilian Celiac Associations (ACELBRA) from Brazilian Federative Units (Ceará, Federal District, Espírito Santo, Maranhão, Minas Gerais, Pará, Paraná, Rio de Janeiro, Rio Grande do Sul, Santa Catarina, São Paulo); and the Brazilian Society of Pediatrics, from all Brazilian regions.

### 2.2. Instrument and Application

The questionnaire used to evaluate FN was previously validated and composed of 25 items [3]. It is a self-applicable questionnaire, answered by one of the child’s caregivers. For this study, questions were added to characterize participants and their children. In this sense, the final questionnaire was composed of 45 questions, including three parts: (1) 9 items—sociodemographic profile: Brazilian region, place of residence (urban area, rural area, Indigenous area, and *quilombola* area), caregiver profile (age, sex, degree of kinship, marital status, and education), and child profile (nationality, sex, age, diagnoses, and family income); (2) 11 items—child’s clinical profile: diagnosis, time since diagnosis, symptoms, and clinical manifestations (questions validated by other instruments aimed at patients with GRD [13,14]; and (3) 25 items—FN profile: general food neophobia (FNger) and food neophobia of fruits (FNfru) and vegetables (FNveg).

The scores for assessing the FN of children with GRD followed the parameters defined by Almeida et al. (2022). Responses were coded to take values from 0 to 4 for each question. Thus, the FNger domain score (with nine items) ranges from 0 to 36; the FNfru domain score (eight items) ranges from 0 to 32; and the FNveg domain score (eight items) ranges from 0 to 32. The total score (FNtot) (25 items) ranges from 0 to 100. Adding the values of each answer, the total FN score is calculated; the higher the values, the higher the FN of the child. The scores classify children’s FN into low, moderate, and high levels, according to [12]. Domain score cut-off points: low—up to 13 points; moderate—from 14 to 21 points; and high—22 points or more. Total score cut-off points: low—up to 40 points; moderate—from 41 to 65 points; and high—66 points or more [15].

### 2.3. Statistical Analysis

The internal consistency of FN and their domains was evaluated by Cronbach’s alpha coefficient, obtained by R-4.3.0 software with the “Psych” package, with values above 0.60 being considered acceptable [16,17].

A Friedman test, followed by Dunn’s post hoc test, was used to compare the three domains of FN. The age groups were initially divided into children aged 4 to 7 (4–7) and 8 to 11 (8–11). An appropriate age for FN intervention is eight years old, as younger children seem to be more likely to change their eating behavior than older children, so the ages were grouped into these two strata [15]. We investigated possible sex and age differences in the classification of FN by the Mann–Whitney test. According to gross family income, salary differences were also analyzed based on the minimum wage (MW) (BRL 1212.00) and neophobia. Respondents were classified into eight groups and compared by Kruskal–Wallis test, followed by Dunn’s post hoc test.

Comparisons between children with and without GRD were performed using the Pearson chi-squared test for age groups and sex variables and the Mann–Whitney test for classification of FN. For all analyses, a significant level of 5% was considered.

## 3. Results

In this study, we have integrated established theories on FN and dietary behavior to frame our analysis, providing a stronger theoretical foundation. These theories help contextualize the observed food avoidance behaviors among children with GRD by explaining how restrictive diets and anxiety around food contamination may reinforce neophobic tendencies. This theoretical approach enhances the depth of our interpretation of FN in the context of GRD, linking behavioral theories with the dietary restrictions faced by these children.

### Sample Characterization

The study sample included 209 CD children’s caregivers, most mothers (n = 192, 91.9%) (Supplementary File—Table S1). Among children (n = 209), most were females (n = 121, 57.9%), with a mean age of 7.2 ± 2.304, and presented celiac disease (n = 175, 83.7%) (Supplementary File—Table S2). The most frequent age of GRD diagnosis was three years (n = 31, 14.8%), followed by six and seven years (n = 25 each, 12% each). Of the total sample, 81.3% (n = 170) follow a gluten-free diet, and 17.7% (n = 37) follow a faulty GFD (Supplementary File—Table S2).

The sample included respondents from all regions of Brazil, with the Southeast region having the most significant number of responses (n = 86) and the North region having the smallest number of respondents (n = 9).

Most respondents have a gross family income range between 4 and 6 MW (24.4%) (Supplementary File—Table S1). Exploring the wage disparities associated with the degree of neophobia based on gross family income, a statistically significant difference is observed in the domain of neophobia related to vegetables (*p* = 0.04). After conducting Dunn’s post hoc test for multiple comparisons, the results revealed that the classification of neophobia was significantly higher (*p* < 0.01) in the group whose family income was classified as “above 15 MW” compared to the group with income “up to 2 MW”.

The results indicate that the complete questionnaire used to assess food FN in Brazilian children with GRD showed excellent internal consistency (α = 0.95; Table 1) [16,17].

Most children presented moderate FN (n = 90; 43.1%), followed by high FN (n = 61; 29.2%) and low FN (n = 58; 27.7%) (Table 2). A statistically significant difference (*p* < 0.001) was observed between the three domains. The general neophobia domain presented the highest degree of neophobia, followed by the vegetable neophobia domain. The fruit neophobia domain revealed the lowest degree of neophobia.

The means in children between four and seven years old in the domains of neophobia towards vegetables were significantly higher than children of the same age group in the domain of fruit neophobia (*p* = 0.03). However, no differences were observed between children aged 8 to 11 years between the two domains (*p* = 0.08) (Table 3).

The Supplementary File (Table S3) demonstrates FN according to children’s sex and age and compares the results of this study to data from children without GRD [12]. The GRD sample presents more females and children between the ages of 8 and 11. Regarding FN, total, and its domains, children with and without GRD do not differentiate.

## 4. Discussion

Food neophobia in children with gluten-related disorders may impair the adoption of a healthy gluten-free diet since it is associated with sensory aversions or fears of negative consequences of eating some products, which may be essential for GRD individuals following a GFD [8]. Neophobic individuals seem to perceive new or unknown foods in a different way than others, potentially rejecting them, mainly when they have a fear of unintentional transgression to the GFD by consuming gluten-cross-contaminated food [8].

Despite the importance of the topic, this study is the first to evaluate FN in Brazilian children aged four to eleven years with GRD using a validated questionnaire in Brazilian Portuguese [12].

The age of four to eleven years is a crucial phase of nutritional development, in which children are transitioning to a greater diversity of foods and, at the same time, may be resistant to new foods. Therefore, FN in this age group can have a significant impact on nutritional status, especially in children with GRD who already have a restricted diet [8]. Considering the factors that may impact the reduction in fruit and vegetable consumption in children, proposing strategies to overcome this tendency becomes essential to improving the nutritional value of children’s diets, promoting an increase in dietary diversity, especially for FN [5].

The lack of significant age differences in FN among children with GRD may be attributed to the persistent nature of FN in restrictive dietary contexts, where exposure to new foods is limited. Studies indicate that without consistent exposure and positive food experiences, FN behaviors can remain stable throughout childhood, especially in environments where safety concerns, such as the risk of contamination in GRD, reinforce cautious eating patterns [18,19]. Additionally, caregivers’ protective feeding practices in managing GRD may further perpetuate FN across different ages, as parents often limit dietary variety to ensure safety [18].

### 4.1. Instrument Reliability

The questionnaire, initially developed for assessing FN in children aged 4 to 11 years without any specific clinical condition, showed evidence of reliability for assessing children with GRD belonging to the same age group [3]. Thus, by estimating the extent of FN in this population, the questionnaire contributes to improving the nutritional interventions to be carried out.

The excellent internal consistency observed in the FN assessment questionnaire in children with GRD is consistent with the parameters established in the literature, which consider α ≥ 0.9 as indicative of excellent reliability [3]. This result is particularly significant, confirming that the questionnaire maintains its precision and comparability even when applied to a specific population, such as children with GRD.

The validity and reliability of the questionnaire reinforce its usefulness in future studies and clinical interventions intended for this group. The recent application of the questionnaire in a particular population also suggests that it can be adapted for other contexts, maintaining its ability to measure FN with high precision [8].

### 4.2. Profile of Caregivers and Children

The female figure as the primary caregiver (95.3%) and knowledgeable about children’s eating habits was similar to the study’s results by De Almeida et al. [20], which evaluated FN in 593 Brazilian children with autism spectrum disorder (ASD). Women and mothers are more concerned about their children’s health and participate more frequently in studies [21,22,23]. In Brazil, in 2019, women spent an average of 21.4 h per week taking care of people and household chores, while men only spent 11 h, which shows that mothers are the main people responsible for caring for children [24], justifying the large number of mothers in our sample. The connection between GRD, dietary restrictions, and FN can be attributed to the necessity of a strictly controlled diet, which limits exposure to a diverse range of foods, thus reinforcing neophobic tendencies. Children with GRD often experience heightened anxiety over food safety due to the risks associated with gluten contamination, which may increase their reluctance to try new foods [25,26]. Additionally, parental feeding practices aiming to prevent dietary transgressions can inadvertently strengthen FN by frequently offering only familiar, safe foods, creating a cycle that further restricts dietary variety and reinforces FN behaviors [22].

The greater participation of girls in the sample is in line with other studies that had CD as an investigation since the prevalence of celiac disease is twice as high among females than males [27], and our sample was most composed of CD among the GRD [27,28]. In the study performed by Setavand et al. (2021), who determined anthropometric indices and clinical indicators in children with CD, 63.7% (n = 230) of participants were female [26]. As observed in the Supplementary File (Table S3), females were significantly the majority among GRD children compared to Brazilian children without GRD.

Most children with GRD presented CD and followed a gluten-free diet. Children with food allergies tend to eat fewer fruits and vegetables [29,30,31] and, often, the worse the allergy, the less variety and quantity of these foods are consumed [29,30,31].

While one might think that children with food allergies would have a greater supply of fruits due to restrictions on other foods, the reality is that these children tend to consume fewer fruits and vegetables [32]. This is because dietary restrictions often lead to a less varied diet, where concerns about possible adverse cross-reactions reduce the introduction of new foods, including fruits and vegetables [33]. Additionally, fear of cross-contamination or allergic reactions may lead parents and caregivers to limit food variety as a safety measure [33].

These individuals may be apprehensive about possible adverse reactions to foods contaminated with gluten, a concern often associated with uncertainty about contamination risks and the safety of gluten-free products [34]. Consequently, individuals with GRD may reject unfamiliar products, including fruits and vegetables [34], due to a lack of familiarity [35].

Faced with dietary restrictions imposed by GRD, parents may feel “pity” or guilt when forcing their children to try new foods or those that were initially rejected [36,37]. This attitude may lead to frequently offering foods that children already like to “lighten” the burden of a restrictive diet instead of introducing new foods [36,37]. This behavior may inadvertently reinforce neophobia, particularly in foods such as fruits, which might be more accepted if the introduction were more insistent and gradual [36]. In addition, fear of contamination and uncertainty about food safety may exacerbate FN, even in families with higher incomes, which theoretically would have access to a more varied diet [5,37].

High levels of dietary restriction can impact children’s cognitive and motor development, as well as their health [38]. It reinforces that FN can harm the nutritional status and health of children with GRD due to a very restricted diet, which can generate dietary monotony [8].

A review between January 1998 and January 2019 that described nutritional deficiencies in CD children on GFD discussed the clinical consequences related to these nutritional imbalances and found that, regardless of whether they followed a GFD or not, children are at risk of consuming a diet rich in fat and insufficient in fiber, iron, vitamin D, and calcium [39]. The authors suggested nutritional education to train CD children, the importance of labels with information about GRD, food choice, and the combination of macro and micronutrients. Also, the increase in natural food intake (fruits and vegetables) does not present a risk of gluten contamination. CD children on a GFD should be encouraged to alternate the intake of pseudocereals with the consumption of commercial gluten-free products that have been fortified or enriched, in addition to using local, naturally gluten-free foods [39].

### 4.3. Prevalence of FN

The findings indicate that children with GRD tend to exhibit a more homogeneous distribution across low, moderate, and high levels of FN, with a higher percentage of moderate FN (43.1%; n = 90). In contrast, in the study by Almeida et al. [12], most Brazilian children in the same age group, without any associated clinical condition, predominantly exhibited low FN (39.9%; n = 333), with a lower prevalence of moderate FN (36.7%; n = 408). The prevalence of high FN was greater in Almeida’s study (33.4%; n = 371) compared to the present study, suggesting that while children with GRD are more likely to exhibit moderate FN, the general Brazilian pediatric population might show a more polarized distribution between low and high levels of FN [40].

Children with high FN may reject new or unfamiliar foods more strongly, exacerbating the challenges of maintaining an appropriate and restrictive diet [12]. This can lead to insufficient intake of essential nutrients, particularly in critical food groups like vegetables, often rejected by children with high FN [12,41]. Such deficiencies may impair growth, development, and overall health while also creating a stressful eating environment that negatively impacts family mealtime dynamics [16,26,30].

The results about the domains (general, vegetable, and fruit neophobia) in the study with Brazilian children are consistent: higher prevalence of general and plant neophobia, with fruit neophobia being lower [12]. The authors reported that the general neophobia domain presented 41.1% high neophobia, vegetable neophobia at 41.7%, and fruit neophobia at 30.2% [12]. This similarity suggests that, in different populations, general and vegetable neophobia tend to be more pronounced, while fruit neophobia is comparatively less common.

Regarding vegetable neophobia, the findings are corroborated by the fact that vegetable consumption is limited and insufficient in the Brazilian general population. On average, Brazilians consume about ten different types of vegetables, which make up roughly 80% of their total vegetable purchases [42]. On the other hand, the lower neophobia observed for children with GRD in the fruit domain represents a contrasting scenario to the fact that, in the general population, the average daily fruit consumption per person is less than 50 g, which is far below the World Health Organization’s recommendation of at least 400 g of fruits and vegetables combined per day [43].

The lower intensity of FN in fruits in the present study, compared to other food groups such as vegetables, may be associated with some factors. Fruits generally have more pleasant and familiar flavors for children, such as sweets, which can ease their acceptance. Furthermore, fruits are often part of the child’s first eating experiences during food introduction, creating early familiarity. Culturally, fruit consumption is encouraged from an early age, which can reduce resistance to these foods [38]. In addition, GRD children have limited access to crackers, cookies, cakes, bread, and other snacks prepared using wheat flour or other gluten-containing ingredients [27]. Gluten-free products are expensive, hard to find, and have poor nutritional, sensory, and technological quality. Therefore, GRD children are expected to have more offers of fruits, mainly in small daily meals, resulting in lower rates of fruit FN.

The analysis of Table 2, which presents the distribution of the sample of children with GRD according to the classification of food neophobia, reveals variations in the distributions of the different levels of FN in the evaluated domains: general FN, FN for fruits, and FN for vegetables. In the domain of general FN, a greater concentration of children was observed at moderate (43.5%) and high (38.7%) levels. In comparison, only 17.7% were classified as having low FN, which indicates a less balanced distribution. The distribution of FN for fruits was more homogeneous, with 36.3% of the children presenting low FN, 31.1% moderate, and 32.6% high. Similarly, in the domain of FN for vegetables, the children were also distributed relatively even between the low (29.2%), moderate (33.5%), and high (37.3%) levels.

The questionnaire’s total score reflects this balanced distribution, with approximately one-third of the children in each FN category (low, moderate, and high). This homogeneous distribution suggests that FN is a phenomenon widely present among children with GRD, without a clear tendency towards a specific level of FN. The significant presence of high FN in approximately one-third of the sample points to potential nutritional challenges, as these children may be more prone to a limited diet, negatively impacting their health and development.

The health benefits and pleasant taste are frequent motivational factors for consuming fruits and vegetables, contributing to maintaining the body’s health and nutritional balance [39]. No statistical difference between age groups was observed in total neophobia or domains. This corroborates data from studies on Brazilian children published by De Almeida et al. and children with ASD [41].

### 4.4. Analysis of the Interaction Between FN and Sociodemographic Factors

The comparative FN analysis between sex and age did not demonstrate a significant relationship.

The findings of De Almeida et al. [12] suggest that this domain-specific neophobic condition is not differentiated by sex. However, another critical aspect is that genes linked to the female sex play a crucial role in young children’s predisposition to avoid unfamiliar foods [44]. This highlights a potential gap in understanding the relationship between sex and FN, a gap that may be addressed by the current study’s observations and findings regarding girls.

Younger children (four to seven years old) tend to present more FN concerning vegetables than fruits due to the more bitter taste and less pleasant textures of vegetables, in addition to less early exposure [45]. In contrast, being sweeter and introduced earlier in the diet, fruits are more accepted [45]. This difference highlights the importance of strategies encouraging the acceptance of vegetables from an early age.

Introducing solid foods in Brazil often begins with fruits, which are naturally sweet and more acceptable to children than vegetables [45]. This pattern may favor greater acceptance of fruits over vegetables with more bitter flavors and less pleasant textures [41]. Studies show that the preference for sweet flavors, such as fruits, is innate and reinforced mainly during childhood eating experiences [46].

Although studies indicate a decrease in FN with age in children without dietary restrictions [38,43], this generalization is not entirely consolidated. The description of these results is essential because FN reduces dietary diversity and limits the consumption of nutrients necessary for maintaining and promoting health. To improve the quality of nutrition throughout life, providing healthier eating habits from birth and knowledge about childhood FN is a crucial aspect of this scenario [46].

The analysis of FN according to gross income links aversion to specific foods, in this case, vegetables, to economic disparity, indicating that families with higher incomes may present more pronounced neophobia concerning this specific food group.

In the study by De Almeida et al. [41] with children with ASD, the monthly family income was low, where 57.5% received up to 3 MW. This differs from the Brazilian study on FN in neurotypical children, where 69.1% had a family income greater than 3 MW [36].

This highlighted a notable disparity in the two populations’ socioeconomic profiles.

Studies by Puleo et al. [45] and Ayoughi et al. [47] conducted to evaluate FN and its determinants in children and adolescents identified that family income has a significant influence on FN among participants and did not find higher levels of neophobia among children from higher-income families. The results indicate that parents with higher incomes tend to impose fewer restrictions regarding weight control and eating strategies for their children. This scenario gives children the freedom and resources to explore a broader range of foods, resulting in greater exposure to diverse food varieties [47]. This finding highlights the complexity of socioeconomic influences on eating attitudes and underlines the importance of considering financial factors when addressing eating behaviors in specific contexts.

The paradox identified in the present study, in which children with GRD and higher income present high neophobia towards vegetables, challenges the assumption that higher income automatically translates into better food choices. This suggests that cultural factors, personal perceptions, and individual experiences can influence food preferences independently of income, revealing the complexity of food choices and the need to consider variables beyond economic access.

The study pioneered the investigation of FN in Brazilian children with GRD using a validated and culturally adapted questionnaire. It is crucial, as it addresses an underexplored area with significant implications for child health and development. This highlights the importance of recognizing limitations, such as sample size and generalization of results, and suggests the need for more longitudinal studies to confirm the findings.

Furthermore, the participation of caregivers as respondents may have caused some bias. However, it is essential to highlight that parents play a crucial role in feeding their children, reinforcing the findings’ significance.

FN in children can significantly impact nutritional development [48]. It is linked to a lower intake of fruits and vegetables, essential for growth and disease prevention [48]. This behavior may lead to a monotonous diet, resulting in nutrient deficiencies, particularly vitamins A, C, and iron [38]. Such deficiencies compromise immune function and cognitive development [31]. The situation worsens when combined with dietary restrictions due to allergies or intolerances [20]. Addressing food neophobia is crucial to reduce these risks and improve dietary quality in children [20].

The presence of diagnoses, such as food allergies and gluten sensitivities alongside GRD, adds complexity to understanding FN in affected children. These conditions impose additional dietary restrictions that may heighten FN by reinforcing avoidance behaviors and limiting exposure to a broader range of foods. For instance, children with multiple food sensitivities may experience increased anxiety around mealtime, which could further entrench neophobic behaviors. Considering these diagnoses allows for a more nuanced interpretation of FN’s impact, as the compounded effect of various dietary limitations can shape distinct neophobic patterns beyond those associated solely with GRD. The nutritional consequences of FN in children highlight the need for early, strategic interventions to promote a balanced diet. Educational strategies for parents and children have proven effective in reducing FN and encouraging healthy food intake. Creating positive food environments and offering new foods regularly can decrease resistance and improve acceptance. This can help prevent nutritional deficiencies and support healthy growth. Early awareness and intervention are key to ensuring a healthier future for these children.

## 5. Conclusions

Based on the presented data, this pioneering study provides essential insights into FN in Brazilian children with GRD. Using a validated questionnaire in Brazilian Portuguese, the study revealed a balanced distribution among low, moderate, and high levels of FN, in general, neophobia, and the specific domains of fruits and vegetables. This homogeneous distribution suggests that FN is widely present among children with GRD, without a clear tendency towards a specific level, highlighting the need for tailored nutritional interventions to minimize the negative impacts of a restricted diet. In the study that evaluated FN in Brazilian children without GRD, high neophobia was more present than in our study.

Furthermore, the study underscores the complexity of food choices influenced by economic, cultural, and individual perception factors. Children with higher incomes exhibited greater neophobia towards vegetables; therefore, higher income does not automatically lead to better food choices. These findings emphasize the importance of considering various factors when addressing FN in children with GRD. Further longitudinal research may be interesting to confirm these findings and explore the associations between FN, family income, and child development, particularly in vulnerable populations such as those with GRD.

To support practitioners working with children affected by GRD and FN, tailored interventions could emphasize gradual food exposure and structured dietary guidance. Practitioners may encourage caregivers to introduce new foods in a safe, positive environment, reducing the anxiety associated with restricted diets. Behavioral strategies, such as positive reinforcement and consistent modeling of varied food choices by parents, could also be beneficial. Additionally, multidisciplinary support involving dietitians, psychologists, and pediatricians may help address the emotional and dietary aspects of FN, fostering healthier, more diversified eating habits in children with GRD, since the clinical and nutritional approach to GRD must transcend the simple exclusion of gluten and incorporate nutritional strategies that address behavioral aspects, such as assessment and approach to FN, promoting adequate nutrition and health.

## Figures and Tables

**Table 1:** Internal consistency of the FN questionnaire by domain in children with GRD (n = 209). Brazil, 2021–2023.

	Number of Items	Cronbach’s Alpha (IC 95%)	Classification
General neophobia	9	0.84 (0.81–0.87)	Good
Fruit neophobia	8	0.93 (0.91–0.94)	Great
Vegetable neophobia	8	0.93 (0.91–0.94)	Great
Total	25	0.95 (0.94–0.96)	Great

**Table 2:** Distribution of the sample according to FN classification in children with GRD (n = 209). Brazil, 2021–2023.

		Food Neophobia		
Domains *	Low	Moderate	High	*p* ***
General ^B^	37 (17.7%)	91 (43.5%)	81 (38.8%)	
Fruits ^A^	91 (43.5%)	66 (31.6%)	52 (24.9%)	<0.001
Vegetables ^B^	61 (29.2%)	70 (33.5%)	78 (37.3%)	
Total questionnaire score **	58 (27.7%)	90 (43.1%)	61 (29.2%)	

* Domain score cut-off points: low—up to 13 points; moderate—from 14 to 21 points; and high—22 points or more. ** Total score cut-off points: low—up to 40 points; moderate—from 41 to 65 points; and high—66 points or more. *** Friedman test. Domains with the same letters do not differ significantly (*p* > 0.05).

**Table 3:** Distribution of the sample and comparison of FN according to gender and age group (n = 209). Brazil, 2021–2023.

	Gender			Age Group		
	Girls	Boys	p *	4–7 y	8–11 y	p *
Tertial Approximation	121	88		87	122	
General neophobia						
Low (≤13)	22 (18.2%)	15 (17.0%)		20 (23.0%)	17 (13.9%)	
Moderate (14 to 21)	59 (48.8%)	32 (36.4%)	0.114	37 (42.5%)	54 (44.3%)	0.123
High (≥22)	40 (33.0%)	41 (46.6%)		30 (34.5%)	51 (41.8%)	
Fruit neophobia						
Low (≤13)	55 (45.5%)	36 (40.9%)		40 (46.0%)	51 (41.8%)	
Moderate (14 to 21)	38 (31.4%)	28 (31.8%)	0.447	31 (35.6%)	35 (28.7%)	0.214
High (≥22)	28 (23.1%)	24 (27.3%)		16 (18.4%)	36 (29.5%)	
Vegetable neophobia						
Low (≤13)	36 (29.8%)	25 (28.4%)		24 (27.6%)	37 (30.3%)	
Moderate (14 to 21)	38 (31.4%)	32 (36.4%)	0.882	29 (33.3%)	41 (33.6%)	0.614
High (≥22)	47 (38.8%)	31 (35.2%)		34 (39.1%)	44 (36.1%)	
Total						
Low (up to 40)	37 (30.6%)	21 (23.9%)		24 (27.6%)	34 (27.9%)	
Moderate (41 to 65)	49 (40.5%)	41 (46.6%)	0.492	41 (47.1%)	49 (40.1%)	0.542
High (66 or more)	35 (28.9%)	26 (29.5%)		22 (25.3%)	39 (32.0%)	

* Mann–Whitney test. Total score cut-off points: low–up to 40 points; moderate—from 41 to 65 points; and high—66 points or more.

## Data Availability

The study did not report any data.

## Supplementary Materials

The following supporting information can be downloaded at: https://www.mdpi.com/article/10.3390/nu16223924/s1, Table S1: Caregivers’ sociodemographic and economic characteristics (n = 209); Table S2: Children’s sociodemographic and health and diet conditions (n = 209); Table S3: Food Neophobia and Children’s Sex and Age according to GRD.
