# Supplementary material for: Dietary Challenges in Children with Gluten-Related Disorders: A Study on Food Neophobia
Source: Nutrients. 2024 Nov 17;16(22):3924. doi: 10.3390/nu16223924 (PMC11597788; doi:10.3390/nu16223924)
Supplement: Supplementary file 1 [file nutrients-16-03924-s001.zip › nutrients-3313237-supplementary.pdf]

Table S1. Caregivers' sociodemographic and economic characteristics (n=209).

Characteristics		Sample (n = 209)	
		**Frequency**	**%**
**Caregivers**			
**Sex**	Female	203	97.13
	Male	06	2.87
**Age**	≤40 y	131	62.68
	>40 y	78	37.32
**Kinship**	Mother	192	91.87
	Father	04	1.91
	Other (sister; grandmother stepmother; aunt; cousin friend;)	13	6.22
**Schooling level**	Elementary School	2	1
	High school	40	19.15
	Undergraduate	21	10
	Graduate	51	24.40
	Postgraduate Degree	95	45.45
**Marital status**	Married/Living in a stable union	175	83.73
	Divorced	12	5.74
	Single	22	10.53
**Income**	<1 minimum wages	16	7.66
	1-2 minimum wages	27	12.91
	3-4 minimum wages	19	9.10
	4-6 minimum wages	51	24.40
	7-9 minimum wages	27	12.91
	10-15 minimum wages	30	14.35
	> 15 minimum wages	20	9.57
	Uninformed	19	9.10
**Residence**	Urban area	199	95.21
	Rural area	10	4.79
**Region**	North	09	4.30
	Northeast	32	15.31
	Midwest	26	12.44
	Southeast	86	41.15
	South	56	26.80

Table S2. Children's sociodemographic and health and diet conditions (n=209).

**Characteristics**		Sample (n = 209)	
		Frequency	%
**Sex**	Female	121	57.90
	Male	88	42.10
**Age**	04-07 y	87	41.63
	08-11 y	122	58.37
**Diagnosis**	Food allergy	37	17.70
	Food allergy and Attention deficit hyperactivity disorder (ADHD)	02	0.96
	Food allergy and Celiac disease	03	1.44
	Celiac disease	28	13.40
	Food allergy and Autism Spectrum Disorder (ASD)	06	2.87
	Food allergy and Food intolerance	26	12.44
	Food intolerance	56	26.79
	Others	11	5.26
	None	40	19.14
**Gluten-related eating disorder**	Wheat allergy	07	3.35
	Dermatitis herpetiformis	05	2.39
	Celiac disease	159	76.08
	Celiac disease and Dermatitis herpetiformis	11	5.26
	Celiac disease, Dermatitis herpetiformis, and Wheat allergy	02	0.96
	Celiac disease and gluten sensitivity	03	1.44
	Non-celiac Gluten sensitivity (NCGS)	18	8.61
	Others	04	1.91
**Time since children's CD diagnosis**	≤1 y	07	3.35
	1–2 y	42	20.10
	3–4 y	45	21.53
	5–6 y	43	20.57
	7–8 y	40	19.14
	9–11 y	32	15.31
**Diagnosis by specialists and/or laboratory tests**	Yes	197	94.26
	No	12	5.74
**Follow the diet for some GRD**	Yes	170	81.34
	Yes, with flaws	37	17.70
	No	02	0.96
**Food restriction (could select more than one option - n =247)**	None	126	51.01
	Milk and/or derivatives	78	31.57
	Egg	10	4.04
	Peanuts	09	3.64

Sugar	08	3.23
Soy	06	2.42
Others*	04	1.61
Artificial colors (G6PD)	04	1.61
	02	0.80
Seafood		

Table S3. Food Neophobia and Children's Sex and Age according to GRD.

Characteristics		With GRD (n = 209)	Without GRD[1] (n = 1112)	p
**Sex**	Female	121 (57.9%)	558 (50.2%)	0.049*
	Male	88 (42.1%)	554 (49.8%)	
**Age**	04-07 y	87 (41.6%)	592 (53.2%)	0.003*
	08-11 y	122 (58.4%)	520 (46.8%)	
**Food Neophobia**	**General neophobia**			
	Low (≤13)	37 (17.7%)	301 (27.1%)	
	Moderate (14 to 21)	91 (43.5%)	354 (31.8%)	0.350**
	High (≥22)	81 (38.8%)	457 (41.1%)	
	**Fruit neophobia**			
	Low (≤13)	91 (43.5%)	451 (40.6%)	
	Moderate (14 to 21)	66 (31.6%)	325 (29.2%)	0.198**
	High (≥22)	52 (24.9%)	336 (30.2%)	
	**Vegetable neophobia**			
	Low (≤13)	61 (29.2%)	296 (26.6%)	
	Moderate (14 to 21)	70 (33.5%)	352 (31.7%)	0.247**
	High (≥22)	78 (37.3%)	464 (41.7%)	
	**TOTAL**			
	Low (up to 40)	58 (27.7%)	333 (39.9%)	
	Moderate (41 to 65)	90 (43.1%)	408 (36.7%)	0.721**
	High (66 or more)	61 (29.2%)	371 (33.4%)	

[1] Source: de Almeida et al. [12] de Almeida, P.C.; Vasconcelos, I.A.L.; Zandonadi, R.P.; Nakano, E.Y.; Raposo, A.; Han, H.; Araya-Castillo, L.; Ariza-Montes, A.; Botelho, R.B.A. Food Neophobia among Brazilian Children: Prevalence and Questionnaire Score Development. *Sustainability 2022, Vol. 14, Page 975* **2022**, *14*, 975, doi:10.3390/SU14020975.

* Pearson chi-squared test;

** Mann-Whitney test.

12. de Almeida, P.C.; Vasconcelos, I.A.L.; Zandonadi, R.P.; Nakano, E.Y.; Raposo, A.; Han, H.; Araya-Castillo, L.; Ariza-Montes, A.; Botelho, R.B.A. Food Neophobia among Brazilian Children: Prevalence and Questionnaire Score Development. *Sustainability 2022, Vol. 14, Page 975* **2022**, *14*, 975, doi:10.3390/SU14020975.
